# A Review on Bioconversion of Agro-Industrial Wastes to Industrially Important Enzymes

**DOI:** 10.3390/bioengineering5040093

**Published:** 2018-10-28

**Authors:** Rajeev Ravindran, Shady S. Hassan, Gwilym A. Williams, Amit K. Jaiswal

**Affiliations:** 1 School of Food Science and Environmental Health, College of Sciences and Health, Dublin Institute of Technology, Cathal Brugha Street, D01 HV58 Dublin, Ireland; rajeev.ravindran@mydit.ie (R.R.); shady.hassan@mydit.ie (S.S.H.); 2 School of Biological Sciences, College of Sciences and Health, Dublin Institute of Technology, Kevin Street, D08 NF82 Dublin, Ireland; gwilym.williams@dit.ie

**Keywords:** agro-industry waste, enzymes, solid-state fermentation, pretreatment, optimization

## Abstract

Agro-industrial waste is highly nutritious in nature and facilitates microbial growth. Most agricultural wastes are lignocellulosic in nature; a large fraction of it is composed of carbohydrates. Agricultural residues can thus be used for the production of various value-added products, such as industrially important enzymes. Agro-industrial wastes, such as sugar cane bagasse, corn cob and rice bran, have been widely investigated via different fermentation strategies for the production of enzymes. Solid-state fermentation holds much potential compared with submerged fermentation methods for the utilization of agro-based wastes for enzyme production. This is because the physical–chemical nature of many lignocellulosic substrates naturally lends itself to solid phase culture, and thereby represents a means to reap the acknowledged potential of this fermentation method. Recent studies have shown that pretreatment technologies can greatly enhance enzyme yields by several fold. This article gives an overview of how agricultural waste can be productively harnessed as a raw material for fermentation. Furthermore, a detailed analysis of studies conducted in the production of different commercially important enzymes using lignocellulosic food waste has been provided.

## 1. Introduction

The food and agriculture (F&A) industry is growing at a rapid pace. The rapidly growing population, along with improving economic growth, has attracted significant investments in the F&A industry, amounting to $75 billion in 2017. With increasing industrialization of the agricultural sector, the resulting waste generation represents a significant environmental challenge. Five million metric tonnes of biomass are produced annually from agriculture [1]. Most of the wastes generated by agro-based food industries are high in nutrients and can form breeding grounds for disease-causing microbes if left unprocessed and inadequately treated. Interestingly, these wastes can serve as raw materials for the production of value- added products or as a source of renewable energy.

New directives by the European Union emphasize the concepts of the ‘Bioeconomy’ and ‘Biorefinery’, whereby the wastes of one industry can serve as the raw material for another [2]. Many wastes generated by agro-industries are lignocellulosic in nature; they are generally high in polysaccharides such as cellulose and hemicellulose and, lignin (an aromatic polymer), in addition to containing other nutrients such as proteins, lipids, pectin and polyphenols. Food waste from households is the most readily available form of waste and there are systems in place for its effective disposal. However, due to its highly heterogeneous nature, the effective utilization of household food waste for certain purposes is impractical. Sustainable industries require a constant source of cost-effective raw materials for efficient functioning. In the first half of the twentieth century, the successful utilization of agricultural waste products as carbon and nitrogen sources for such processes as antibiotic fermentation established an important precedent. Linking waste streams from certain industries to agro-based businesses for effective valorisation will contribute to solving the problem of waste accumulation. This calls for practical valorisation studies in the area of food waste utilization for the production of value-added products [3].

## 2. Agro-Industry Wastes

One third of the food (approximately 1.3 billion tonnes) produced globally for human consumption is lost or wasted every year. Fruits and vegetables, plus roots and tubers have the highest wastage rates of any food, representing 40–50% (520–650 million tonnes) of the global quantitative food losses and waste per year. In the EU, food waste amounts to 89 million tons of food per year (39% of this food waste occurs during manufacturing processes), while total agricultural residue production (crop residues, or parts of cropped plants that are not consumed as food) in the EU amounts to 367 million tons per year [4]. However, much of the residue is consumed at the farm level for use as animal bedding and fodder, and various horticultural uses.

## 3. Valorisation of Agro-Industry Wastes

Several studies have been conducted over the past few decades examining the effective utilization of agro-industry waste as a potential raw material for the production of value- added products. Most of these studies have been conducted in countries whose economies are heavily reliant on agriculture. For example, Brazil is the largest producer and exporter of sugar cane, and unsurprisingly, the second largest producer of bioethanol in the world [5]. Currently, bioethanol and related liquid-fuel- based production are the only commercial operational processes that utilize agro-industry waste as raw materials. This is achieved by following fermentation strategies such as hydrolysis of waste followed by fermentation or simultaneous saccharification and fermentation [6]. Nonetheless, researchers began looking at viable options to convert lignocellulosic residues that arise from agriculture as a raw material for the production of various enzymes [7]. Such wastes are highly rich in polysaccharides (for example, cellulose, hemicellulose, starch, pectin, and inulin; Table 1).

## 4. Industrial Enzymes

Enzymes are biological catalysts that find applications in several industries that range from baking and brewing to paper, pulp and the detergent industry. Due to their high degree of substrate specificity, and stringent yet robust operational parameters, they are preferred over chemical catalysis in many situations. Enzymes are found in all living systems. The advent and proliferation of recombinant DNA technology has enabled scientists to clone and mass produce enzymes of any origin in microbes to fit the demand of various industries. Strain improvement via mutation is another technology widely employed in industry to increase the yield of enzyme production [7].

The global market for industrial enzymes is expected to increase at a compounded annual growth rate (CAGR) of 4.7% between 2016 and 2021 (growing from approximately $5.0 billion in 2016 to $6.3 billion in 2021 [17]). Despite the tangible demand, enzymes are relatively expensive reagents, and this adds to the operational cost of processes that utilize them. Critical analysis of process plant economics for enzyme production reveals that almost 50% of the cost of production is associated with capital investment, while the cost of raw materials accounts for almost one third of such costs. Substitution or complementation of feedstocks with lignocellulosic sources can result in an increased return on investment. Table 2 represents the cost of a few commercial enzymes that act upon lignocellulosic plant biomass.

### 4.1. Agro-Industry Wastes as Substrate

Recent studies involving enzyme production using agro-industry wastes have primarily investigated the effect of pre-treatment measures in influencing the yield. Pre-treatments are techniques widely used in production processes involving lignocellulose-based raw material where the complex plant structure is disrupted by physical, chemical or a combination of methods to improve digestibility [19,20]. An interesting study conducted by Salim, et al. (2017) deployed a new strain of Bacillus to study the production of four enzymes (α-amylase, protease, cellulase and pectinase) using different agricultural residues as growth substrates (soybean meal, sunflower meal, maize bran, maize pericarp, olive oil cake and wheat bran) [21]. Each residue was subjected to different pre-treatment measures (acid/alkali, ultrasound and microwave treatments) to determine their effect on enzyme production. Chemical pre-treatments were found to be superior over other techniques, with highest yields of protease and amylase achieved using alkali -treated corn pericarp. Another study conducted by Leite et al. (2016) studied the application of ultrasonication as a potential pre-treatment for olive pomace to improve enzyme yield using solid-state fermentation [15]. The authors reported a 3-fold increase in xylanase activity and a 1.2-fold increase in cellulase activity employing *Aspergillus niger* as the producer microbe.

While media formulations based on agricultural wastes are heterogeneous by nature, studies have been conducted on optimization to improve yield. Gustavo et al. (2017) conducted an extensive study to optimize a solid-state fermentation process involving different agro-industrial wastes and *Rhizopus microsporus var. oligosporus* as the producer microbe [22]. The application of four lignocellulosic substrates viz. wheat bran, wheat flour type II, soy bean meal and sugarcane bagasse were assessed in their study as potential carbon sources for amylase production. Their findings suggested that the best individual substrate for amylase production was wheat bran.

### 4.2. Production of Enzymes at Industrial Scale

#### 4.2.1. Enzymes that Act on Polysaccharides

##### α-amylase

Alpha amylases (endo-1, 4-α-d-glucanglucanohydrolase EC 3.2.1.1) are a family of enzymes that randomly cleave α-1, 4 linkages between adjacent glucose subunits in polysaccharides, resulting in the release of short chain oligomers and α-limit dextrins. Alpha amylases find a wide range of applications, spanning manufacturing processes for detergents, bread, beer textiles, paper, pulp and pharmaceuticals [23]. Alpha amylases are industrially produced via submerged fermentation using (often genetically improved) *Bacillus* and *Aspergillus* species, but the potential of solid-state culture has been emphasized in the literature [24]. A large spectrum of bacterial and fungal species has been found which produce alpha amylase enzymes possessing different characteristics such as thermos-stability, halo-tolerance, psycho-tolerance and alkali-stability [25,26,27].

With a view to decreasing the production cost, much research has been dedicated to using lignocellulose as a carbon source for amylase production. Early studies featured banana waste (pre-treated banana fruit stalk by autoclaving at 121 °C for 60 min) as a substrate for the production of high titres of α-amylase (a minimum of 5,345,000 U mg^−1^ min^−1^) using *B. subtilis* CBTK 106 [28]. Francis et al. (2003) conducted optimization studies for the production of α-amylase using spent brewer’s grain (initial moisture of 70%) as substrate (approximately 20% increase in enzyme yield was observed) [11]. *Aspergillus oryzae* was used (inoculum rate of 1 × 107 spores/g dry substrate) as the producing microbe in solid-state culture (incubation temperature of 30 °C). Rajagopalan and Krishnan (2008) were able to achieve a maximum α-amylase enzyme activity of 67.4 U mL-1 by adding sugarcane bagasse hydrolysate (prepared by acid hydrolysis) to the nutrient medium using catabolite de-repressed *Bacillus subtilis* KCC103 as the enzyme producer [29].

##### Amyloglucosidase

Amyloglucosidases (AMG; E.C. 3.1.2.3), also known as glucoamylases, can cleave the α-1,4 linkages found in starch to release glucose molecules. It is an exoamylase, releasing β-D glucose from the non-reducing ends of amylose, amylopectin and glycogen [30]. Amyloglucosidase also breaks α-1,6 glycosidic bonds, but at a slower rate [31]. These enzymes exhibit optimum activity at a pH range of 4.5–5.0 and a temperature range of 40–60 °C [32]. AMG finds applications in the food, brewing and bakery industry [33]. *A. niger* and *A. oryzae* are common strains that are used by industry for the production of commercial AMG [31,34]. However, *Bacillus* sp., *Rhizopus* sp. and *Saccharomyces* sp. have also been reported to synthesise AMG [35].

Several lignocellulose residues have been used as an alternate carbon source for the production of AMG. Using *A. niger* in solid-state fermentation conditions, and wheat bran as substrate, a 5–6-fold increase in enzyme yield was obtained, with yields of 520–560 IU enzyme per gram dry substrate [36]. Pandey et al. (1994) reported improved yields in the production of AMG using *A. niger* growing on rice bran in solid-state culture, supplemented with additional nitrogen sources, and achieving a yield of 339 IU/g of dry substrate [9]. Agri-residues such as cassava, yam, banana (and peels) and plaintain have also been successfully employed as alternate carbon sources for the production of fungal AMG [13].

##### Cellulase

The depolymerisation of cellulose into component glucose molecules requires a combined hydrolysis by three key enzymes: endoglucanase (E.C. 3.2.1.4), exoglucanase or cellobiohydrolase (E.C. 3.2.1.176) (E.C. 3.2.1.91) and β-glucosidase (E.C. 3.2.1.21). They are categorized in the glycoside hydrolase family and catalyse the cleavage of glycosidic bonds [37]. Cellulases are enzymes of great commercial importance, especially because of the key role they play in bioethanol production [38]. In addition to biofuels, cellulases find application in industries ranging bread, brewing, textiles, detergents, paper and pulp sectors [39]. A wide range of bacterial and fungal species produce cellulase enzymes. Commercial fungal cellulases most commonly are produced by improved strains of *Trichoderma reesei* [40]. Examples of other fungal organisms that produce cellulases are *Schizophyllum commune*, *Melanocarpus* sp., *Aspergillus* sp., *Penicillium* sp., and *Fusarium* sp. [37]. In bacteria, cellulases are found in the form of large, extracellular aggregates which are called cellulosomes [41]. Some of the bacterial species which produce cellulosomes include *Clostridium thermocellum*, *Bacillus circulans*, *Proteus vulgaris*, *Klebsiella pneumonia*, *Escherichia coli* and *Cellulomonas* sp. [42].

Owing to its influence on the economics of bioethanol production, the development of cheaper methods to produce cellulases has been a key focus of researchers over the past two decades [43]. Several studies have been conducted to test the efficacy of lignocellulosic food waste as a suitable carbon source for cellulase production. Sun et al. (2011) evaluated the feasibility of using banana peel for cellulase production by *Trichoderma viride* GIM 3.0010 in solid-state fermentation [44]. They reported that banana peel provided the nutrients necessary for cell growth and cellulase synthesis (Carboxy methyl cellulase sodium activity (CMCase) obtained was 10.31 U/gds, respectively; When banana peel was used (the moisture content of 65%) with the inoculum size of 1.5 × 109 spore/flask and incubated at 30 °C for 144 h). In another study, an optimized media was formulated using mango peel as carbon source for cellulase production by *Trichoderma reesei* [45]. *Trichoderma reesei* QM9414 was used as the enzyme producer in a study involving brewer’s spent grain as the substrate [46]. The effectiveness of apple pomace as a carbon source for the production of cellulase was evaluated by Dhillon et al. (2012) [14]. Lactose was added as an additional carbon source. After 48 h of fermentation a high carboxymethyl cellulase activity of 172.31 IU per g of dry substrate was observed.

##### Xylanase

Xylanases (E. C. 3.2.1.8, 1,4-β-xylanxylanohydrolase) are enzymes that break down xylan which is an integral part of plant polysaccharide. Xylan is a complex polysaccharide made of a xylose-residue backbone, with each subunit linked by a β-1,4-glycosidic bond. The xylan backbone can be branched with D-glucuronic acid or D-arabinofuranoside [47]. Xylanases are produced by several bacterial and fungal species. Filamentous fungi that synthesise this enzyme are of particular interest because they secrete it into the media in large quantities in comparison to bacteria [48]. Xylan, being a complex polysaccharide, requires a consortium of enzymes for total hydrolysis. This enzyme system consists of endoxylanases, β-xylosidases, ferulic acid esterase, p-coumaric acid esterase, acetylxylan esterase and α-glucuronidase. Endoxylanases and β-xylosidases are the most extensively studied components of this system [49]. Xylanases have wide applications in industries as diverse as food, biomedical, animal feed and bioethanol [47,50,51].

The suitability of lignocellulosic food waste as a carbon source for the production of commercial xylanases has been studied by many researchers. Lowe et al. (1987) investigated wheat straw as a viable carbon source for the production of a xylanase derived from an anaerobic rumen fungus and achieved high levels of activity (0.507 IU/mL) [52]. *A. niger* and *A. terreus* strains were employed on moistened wheat bran by Gawande and Kamat (1999) to attain 74.5 IU mL-1 of xylanase activity [53]. In another study, grape pomace was used as the substrate for the production of xylanase using *A. awamori* as the enzyme producer [54]. Highest xylanase activity (40 U/g) was achieved after 24 h of incubation time. Addition of 6% glucose as an additional carbon source increased xylanase production significantly. Apple pomace, melon peel and hazelnut shell were used by Seyis and Aksoz (2005) for xylanase production from *Trichoderma harzianum* 1073 D3, achieving a maximum activity of 26.5 U/mg [55].

##### Inulinase

The importance of inulinase has arisen from the emergence of fructose and fructo-oligosaccharides as consumer-preferred sweeteners compared to sucrose in the food and pharmaceutical industry due to their preferred appeal to the human palate. Inulinase acts upon inulin, which is a polyfructose (fructan) chain terminated by a glucose molecule. The fructose units in inulin are bonded together by a β-2,1-linkage [56]. Commercially, fructose syrup is produced from starch by the combined activity of α-amylase, amyloglucosidase and pullulanase, followed by glucose isomerase which converts glucose to fructose. However, the best yield that can be obtained from such a process is 45% of fructose, 50% of glucose, with the remainder being oligosaccharides. The activity of inulinase results in the complete conversion of the substrate to fructose [57]. Inulinase also finds application in the production of bioethanol, citric acid, butanediol and lactic acid.

Inulinases can be sub-classified into exo-inulinases (β-d-fructanfructohydrolase, EC 3.2.1.80) and endo-inulinases (2,1-β-d-fructanfructohydrolase, EC 3.2.1.7) depending upon their modes of activity [58]. Several bacterial and fungal species such as *Streptococcus salivarius*, *Actinomyces viscosus*, *Kluyveromyces fragilis*, *Chrysosporium pannorum*, *Penicillium* sp. and *Aspergillus niger* have been known to synthesise inulinase [59]. The production of inulinase using lignocellulosic substrates has been investigated by many researchers. Gupta et al. (1989) reported that when *Fusarium oxysporum* is grown on the aqueous extracts of roots of *Cichorium intybus*, it produces extracellular inulinase which can be useful for the production of fructose [60]. A newly isolated *Saccharomyces* sp. from spontaneously fermented sugar cane synthesised inulinase when grown on substrates such as banana peel, wheat bran, rice bran, orange peel and bagasse [10]. Coconut oil cake was used in a study which involved the media optimization for inulinase production by employing *Pencillium rugulosum* (MTCC-3487) [61]. Sugar cane baggase and yacon have also been used as substrates in different studies for inulinase production [62,63].

##### Hemicellulases

Hemicellulases are a group of enzymes that attacks the β-1,4-glycosidic bonds which forms the backbone of hemicellulose in lignocellulosic biomass. Hemicellulose is a complex polymeric fraction forming a key component in lignocellulose. Unlike cellulose it is inherently amorphous in nature and is made by a heterogenous group of component sugars which include but are not limited to glucose, xylose, mannose, arabinose and galactose. The component sugars in hemicellulose may vary depending upon the source of the plant biomass [19]. Additionally, sugar acids such as ferulic acid also forms the components of hemicellulose [64]. Hemicellulases are equipped with functional modules that are capable of digesting glycosidic bonds as well as esterified side chain groups. Acetyl and feruloyl esterases hydrolyse acetate or ferulic acid side groups in the plant cell wall structure. Some of the most common hemicellulases that act upon glycosidic bonds include α-glucuronidases, α-arabinofuranosidases, α-d-galactosidases, and mannanases [65].

##### Mannanase

Mannanases are a group of enzymes that degrade mannan, which is an integral part of the plant cell wall. Mannan is a representive of hemicellulose which is found in plants [66]. Three major enzymes are involved in the degradation of linear mannans: 1,4-β-d mannohydrolases or β-mannanases (EC 3.2.1.78), 1,4-β-d mannopyranoside hydrolases or β-mannosidases (EC 3.2.1.25) and 1,4-β-d glucoside glucohydrolases or β-glucosidases (EC 3.2.1.21) [67]. β-mannanases demonstrate endo-hydrolysis activity by cleaving the internal glycosidic bonds, resulting in the release of short chain β-1,4-manno-oligosaccharides [68]. On the other hand, β-mannosidases possess exo-hydrolase activity, attacking the mannan polymer at the non-reducing terminal and degrading mannobiose to individual mannose units [69]. β-glucosidase activity results in the excision of β1,4-glucopyranose units from the non-reducing terminal ends of oligomers released from glucomannan and galactoglucomannan hydrolysis [70]. Several *Bacillus* spp., including different *B. subtitlis* strains, have been reported to produce several mannan- degrading enzymes. Among fungal organisms, many *Aspergillus* spp. have been found to produce mannanase [71]. Other bacterial and fungal species such as *Clostridium* spp., *Pencillium* spp. and *Sterptomyces* spp. have also been known to synthesise mannanase [67].

Due to its ability to effectively remove hemicellulose, mannanase has sparked an increasing interest within the paper and pulp industry [72]. Mannanases also find applications in the food, oil, feed and textile industries [73,74,75]. Many researchers have studied the production of mannanase using lignocellulose and agro-food industry wastes as a substrate. Yin et al. (2013) used a mixture of apple pomace and cottonseed powder as a raw material to produce β-mannanase by *Aspergillus niger* SN-09 in solid-state fermentation [76]. On optimization of process conditions an activity of 561.3 U/g was attained, which was 45.7% higher than that observed in basal medium. The efficacy of mixed substrate fermentation for mannanase production was investigated by Olaniyi et al. (2014) using enzyme producers *Penicillium italicum* and *Trichosporonoides oedocephalis* [77]. They experimented with lime, grape, tangerine and sweet orange peels in different concentrations and reported that mixed substrate fermentation resulted in appreciable enzyme activity. Fermented palm kernel cake was used in another study as the substrate for mannanase production by growing *Aspergillus terreus* SUK-1 in a column reactor [78]. Sugar cane pulp, soy bean mea, locust bean gum and peels from plantain, mango, potato and passion fruit have also been reported to be excellent choices of raw material for the production of β-mannosidase and β-glucosidases [69,79,80].

##### Lactase

Lactase, otherwise known as β-d-galactohydrolase (EC 3.2.1.23), hydrolyses lactose into glucose and galactose [81]. Lactose is the sugar which is found in mammalian milk. Humans produce this enzyme only as infants and this ability lessens as they grow older which leads to lactose intolerance [82]. Hence, lactase is a very important enzyme in the dairy and food industry. Industrial lactase is produced by employing selected strains of *Kluyveromyces lactis*, *Kluyveromyces fragilis*, *A. niger and A. oryzae* [83,84,85,86]. However, several articles can be found that report other organisms that produce lactase. *Candida pseudotropicalis* has been reported to produce lactase when grown on de-proteinized whey [87]. *Trichoderma viride* ATCC 32098 produces a highly thermostable lactase enzyme which is shows 90% activity in a pH range of 3.0–7.5 [88]. Macris (1981) reported the production of extracellular lactase from *Fusarium moniliforme* using wheat bran solid medium. The addition of agricultural by-products such as molasses and whey increased enzyme yield [89].

A few studies have been done to increase the economic feasibility of lactase production by the addition of media components that are cheaply available and lignocellulosic in nature. A *Lactobacillus acidophilus* strain isolated from fermented ragi was reported to produce lactase enzyme, even when the lactose content in the media was reduced to 0.75%, supplemented with 1% ragi [90]. However, lactose was found to be the best carbon source for lactase enzyme production. This may be due to the fact that lactase production is induced by the presence of lactose in the media. A study conducted by Mustranta et al. (1981) employed a mutant strain of *A. niger* for lactase production and attained three-fold increases in enzyme yield [91]. They observed that the mutated strain of *A. niger* produces high levels of lactase enzyme in a medium supplemented by wheat bran. N-methyl-N-nitro-N-nitroso-guanidine (NTG) was used as the mutagenic agent.

##### β-glucanase

1,3-1,4-β-glucans are polysaccharides made up of glucose units that are found in endosperm cell walls of cereals such as barley, rye, sorghum and oats [92]. Endo-1,3-1,4-β-glucanases are enzymes that can hydrolyse the β-1,4 glycosidic linkages at the non-reducing ends of glucans to release cellobiosyl-d-glucose and 3-O-β-d-cellotriosyl-d-glucose. This enzyme is particularly important in the brewing industry. β-glucanases, along with a consortium of xylanase and cellulose, facilitate the reduction of viscosity in fluids with higher solids content. This leads to less water consumption during production processes, saves energy and eliminates the need for several raw materials [93]. β-glucanases also find application in the wine industry, along with pectinases, by facilitating a smooth and fast filtration process, and also increasing the quality of the product [94].

Several microorganisms have been tried and tested for the production of β-glucanase using lignocellulosic industry waste as substrate. *Rhizomucor miehei* CAU432 was grown under solid-state fermentation conditions using oat meal as the sole carbon source to obtain a β-1,3-1,4-glucanase activity of 20,025 U g–1 dry substrate, which was the highest ever reported [95]. Irshad et al. (2012) reported the characterization of exo 1,4-β glucanase from *Tricoderma viride* MBL by culturing the organism in solid-state fermentation using orange peel waste as substrate [96]. *Fusarium oxysporum*, *Bacillus subtilis*, *Penicillium echinulatum*, *Sclerotium rolfsii* have also been reported to produce high levels of endo and exoglucanase when grown on biomass sources [93,97,98,99].

##### Invertase

Invertase, technically known as β-fructofuranosidase (EC.3.2.1.26), is a glycoprotein which catalyses the hydrolysis of sucrose to glucose (dextrose) and fructose. Invertase exhibits optimum activity at a pH of 4.5 and temperature of 55 °C. Saccharomyces cerevisiae is the chief microbe used in the production of invertase enzyme in industry [100]. Invertase is used for the production of invert sugar, which was previously performed by acid hydrolysis. Acid hydrolysis of sucrose results in the 50% conversion of sucrose to invert sugar. Moreover, the acid hydrolysis product also contains impurities whose formation cannot be controlled during inversion. The use of invertase results in 100% inversion of sucrose without the formation of impurities [101]. Since invertase is a commercially expensive enzyme, many researchers have attempted to use cheap carbon sources for its production. Hang and Woodams (1995) tested the efficacy of apple pomace as a potential carbon source for the production of invertase from *Aspergillus foetidus* NRRL 337 [102]. Rashad and Nooman (2009) used red carrot jam processing residue as a medium for solid-state fermentation to characterise invertase synthesised by *S. cerevisiae* NRRL Y-12632 [103]. Bagasse waste, wheat bran and peel waste from orange, pineapple and pomegranate have all been tested and found to be excellent carbon source substitutes for invertase production employing different microbial species [8,104,105].

##### Pectinase

Pectinases are a class of enzymes that catalyse the disintegration of pectin-containing compounds. Pectin compounds are an integral part of the plant cell wall. Pectinases can be classified into two groups according to their mode of action. Pectin esterases catalyse the de-esterification of methyl groups found in pectin to produce pectic acid. Depolymerase enzymes cleave the glycosidic bonds found in pectic acid to release various simpler compounds based on their mode of enzyme action. Protopectinases solubilize protopectin into highly polymerized forms of soluble pectin [106]. Pectinases are used in the fruit juice industry and wine making for clarification and removal of turbidity in the finished product. They also intensify the colour in the fruit extract, while aiding in stabilization and filtration [107].

Many agricultural residue varieties have been tried-and-tested for the production of pectinases using different microbial species. Apple and grape pomace have been extensively used in studies to determine their efficacy as suitable substrates for pectinase production [54,108]. A novel approach was devised by Kashyap et al. (2003) in which they supplemented the fermentation media with Neurobion^®^ tablets (a multi-vitamin) and polygalacturonic acid, while producing pectinase using wheat bran as the carbon source [109]. Maximum enzyme activity was reported to be 8050 U/g dry substrate. A mixture of orange bagasse and wheat bran in the ratio (1:1) was employed as media for pectinase production using the filamentous fungus *Penicillium viridicatum* RFC3 and yielded highest enzyme activities of 0.7 and 8.33 U mL^−1^ for endo- and exo-polygalacturonase and 100 U mL^−1^ for pectin lyase on performing the fermentation process in polypropylene packs [110]. Seedless sunflower heads and barley spent grain are lignocellulosic food processing wastes have also been studied as potential media additives for pectinase production [111,112].

Pectinases have also been produced using a mixture of wastes. Biz et al. (2016) developed an ingenious method to produce pectinases employing citrus peel and sugar cane bagasse in solid-state fermentation mode while avoiding overheating problems [16]. A pilot-scale packed bed reactor was used for this purpose. A uniform pectinase activity of 34–41 U/mL was obtained throughout the bed. A combination of citrus peel and bagasse ensured temperature control while avoiding problems such a bed shrinkage and agglomerate formation since sugarcane bagasse is highly porous in nature. *Aspergillus oryzae* was used as the fermentative microbe.

#### 4.2.2. Enzymes that Act on Proteins

##### Protease

Proteases (EC 3.4.21.62) are enzymes that perform proteolysis by hydrolysing the peptide bonds that link amino acids together in polypeptide chains. Proteases are among the most important hydrolytic enzymes and have been studied since the birth of enzymology. They have always been to the forefront, not only because of the active roles they play in cellular metabolism, but also because of their application in industry. Proteases dominate the total enzyme sales, with a market share of almost 60% [113], and a major component of this is represented by detergent proteases; the latter have a unique ability to remain stable and active at the alkaline pH of the wash environment [114].

Serine proteases, especially subtilisin A, Neutrase^®^ and trypsin are some of the commercially important proteases. Most of the proteases applied in industry are produced by genetically modified strains of *Bacillus* and *Aspergillus* [115,116]. However, much literature is available on the production of proteases by other microbial species that are thermostable and can withstand pH variations. Interestingly, lignocellulosic substrates have been used as raw materials in several studies involving protease production. Nascimento et al. (2011) found that using brewer’s spent grain as the sole carbon source and corn steep liquor as a supplementary nitrogen source for proteinase production by *Streptomyces malaysiensis* AMT-3 was an economically feasible strategy [117]. *Aspergillus* sp. have thoroughly been studied for the production of proteases on different lignocellulosic substrates owing to its filamentous morphology, which enables it to flourish well on solid substrates. Chancharoonpong et al. (2012) studied the growth of *A. oryzae* and the associated protease production in solid-state culture (soybeans) [118]. They obtained the highest protease activity of 84.38 U/g of dry weight after 48 h, coinciding with sporulation. Other substrates such as tomato pomace and Jatropha seed cake have also been used for protease production [119,120].

##### Transglutaminase

Transglutaminases (E.C. 2.3.2.13, protein-glutamine-γ-glutamyl transferase) are a class of transferase enzymes which catalyse the formation of isopeptide bonds between the γ-carboxyamide groups of glutamine residues and the γ-amino group of lysine residues [121]. Transglutaminases are produced in mammalian muscle tissues, as well as microbial cells. However, this enzyme is industrially produced by employing superior strains of *Streptoverticillium mobaraense*, as it is calcium independent and lower in molecular weight compared to other isozymes [122]. The presence of calcium in media formulations leads to the deterioration of industrial equipment by forming calcium oxalate which results in the blockage of pipes, and heat exchangers [123]. Two fungal species viz. *Pythium* sp. and *Phytophthora* sp. have sparked interest in researchers as potential organisms for the manufacture of transglutaminases. These organisms belong to the class Oomycetes and can achieve unprecedented high levels of enzyme expression [124]. Several organisms have been tested as hosts for the over- production of recombinant transglutaminase, including *Escherichia coli*, *Corynebacterium glutamicum* and *Streptomyces lividans* [125].

Transglutaminases find various applications in industry. They are used in products such as flour, baked goods, processed cheese/milk/meat/and fish, cosmetics, gelled food products and leather finishing [124]. Treating wool with transglutaminase after undergoing protease treatment increases the strength, and in turn, the longevity of the wool fibres [126]. Food waste and agro-wastes have been investigated by researchers as potential media components for the production of transglutaminase. Several industrial residues, such as untreated corn grits, milled brewer’s rice, industrial fibrous soy residue, soy hull, and malt bagasse were used by De Souza et al. (2008) as substrates for transglutaminase synthesis by using *Bacillus circulans* BL32 strain in solid-state culture [127]. They reported that the fibrous soy residue, which is rich in protein and hemicellulose, was best suited for enzyme production using *B. circulans* BL32. *Streptoverticillium ladakanum* NRRL-3191 was the enzyme producer in another study where the microbe was grown on media made from hydrolysates of sorghum straw [128].

#### 4.2.3. Other Industrially Important Enzymes

##### Lipases

Lipases find a wide variety of applications owing to the type of reactions they can catalyse. In a living system, lipases facilitate the breakdown and mobilization of lipids within the cells. Within the spectrum of reactions, lipases catalyse some of the industrially interesting processes, including hydrolysis, transesterification, alcoholysis, acidolysis, aminolysis and esterification. They can hydrolyse fats into fatty acids and glycerol at the water-lipid interface and can catalyse the reverse reaction in non-aqueous media. All of these features make lipases a widely sought out enzyme in industries such as detergents, oil, dairy, pharmaceutical, bakery, biopolymer synthesis, biodiesel production and the treatment of fat-containing waste effluents [129]. Lipases from two species viz. *Thermomyces lanuginosus* and *Rhizomucor miehei* are extensively used for various industrial purposes in soluble as well as immobilized form, the latter owing to low stability and difficulty in enzyme-product separation [130,131]. However, other microbial species such as *Bacillus*, *Serratia*, *Psuedomonas* and *Staphylococcus* have also been reported to produce lipases [132].

Lipases can easily be produced by using lignocellulose and waste derived from other sources using different microbes. A study investigated *A. niger* as the enzyme producer for lipase synthesis using peanut cake as substrate along with 1.0% (*v*/*w*) Tween-80, 0.35% (*w*/*w*) (NH4)_2_SO_4_ and 0.40% (*w*/*w*) Na_2_HPO_4_ [133]. An enzyme yield of 49.37 U/g was obtained. Furthermore, using sheanut cake for lipase production reduced the inherent tannin and saponin content, rendering it fit for use as animal feed. Coconut oil cake was used in another study for the optimization of production of two lipolytic isolates (COM-4A and COM-6B) from *Staphylococcus pasteuri*. The newly isolated enzyme COM-4A showed optimum activity at pH of 9.0 and temperature of 50 °C and could be considered for applications in detergent formulations and in biodiesel production [134].

##### Phytase

Phytases are important enzymes in the animal feed and nutrition industries. They were discovered by Suzuki et al., in 1907 while performing the hydrolysis of rice bran [135]. Phytases essentially degrade phytic acid, which is a phosphate storage compound found in grains and seeds. They can be classified into three types according to the position of the phosphate groups they hydrolyze (3-phytase, 4-phytase and 5-phytase) (E. C. 3.1.3.8, E. C. 3.1.3.26 and E. C. 3.1.3.72) [136]. Monogastric animals that consume grains are unable to digest phytic acid due to little-or-no production of phytase. Substances that are used for animal feed, such as oat meal and wheat bran, are rich in phytic acid which is excreted undigested, and thus minerals such as calcium and phosphorus need to be added as supplements Treating animal feed with phytases eliminates the need for addition of minerals and increases nutritive value, while also reducing the phosphate concentration in effluents from pig and poultry farms, and thereby reducing eutrophication potential [137].

*Aspergillus niger* has traditionally been used for commercial production of phytases since 1991. However, several studies have focused on the production of phytases using different microbial species, via both solid-state and submerged fermentation methods. Lignocellulosic substrates have been found to enhance enzyme production when used as media additives. Papagianni et al. (1999) reported that the addition of wheat bran to the fermentation media enhanced *A. niger* growth as well as enzyme production in both solid-state and submerged fermentation production systems [138]. Agri-based residues were utilized for phytase production employing *Nocardia* sp. MB36 as the fermentative microbe by Bajaj and Wani (2011) [139]. They isolated a thermostable phytase which showed activity in acidic and alkaline pH ranges. The microbe was cultured in media supplemented with wheat bran and FeSO4. Orange peel flour was used in another study for phytase production employing *Klebsiella* sp. DB-3FJ711774.1 under submerged fermentation cultivation conditions; the phytase exhibited both thermo- and acid-stability [140].

##### Laccase

Laccases (EC 1.10.3.2) are classified in the multi-copper oxidase family, and catalyse the oxidation of phenolic compounds with the help of molecular oxygen [141]. They were first isolated from the lacquer tree *Toxicodendron vernicifluum* and have since been found in over 20 bacterial species [142]. However, fungal laccases are more abundant in nature, and wood rotting fungi in particular. While bacterial laccases are periplasmic in nature, fungal laccases are extracellular. Three kinds of fungal organisms produce laccase enzyme viz. white rot fungi, brown rot fungi and soft rot fungi [143]. Out of the three, white rot fungi have been found to be the most efficient lignin- degrading microbes [144]. Laccases are commercially important enzymes due to their ability to degrade phenolic and non-phenolic lignin along with recalcitrant pollutants. They are used for the decolourisation and synthesis of dyes, bio-bleaching, baking, bio-pulping, degradation of xenobiotics and effluent treatment [145].

Glucose, fructose, mannose, lactose and maltose are the commonly used carbon sources for the production of laccase. The excess of monosaccharides has a detrimental effect on laccase production n and thus a polymeric substrate like cellulose is a necessary media component [146]. The addition of lignocellulose to culture media has resulted in the increase in laccase activity by several fold. Laccase production employing the white rot fungi, *Trametes versicolor* (CBS100.29), and using barley bran resulted in a maximum enzyme activity of 639 U/L, which was ten times higher than that observed in cultures without lignocellulose [147]. Using ground nut seeds for laccase production also resulted in similar increases in enzyme activity [148]. Other lignocellulosic biomass which has been reported as suitable carbon sources for laccase production include banana skin, ground nut shell, spent coffee ground, rice straw and wheat bran flakes [141,144,149].

#### 4.2.4. Cellulosomes: The Future Prospect of Cellulytic Enzymes

Many aerobic microorganisms rely on endoglucanases, exoglucanases and ancillary enzymes to digest lignocellulosic plant biomass in a synergistic manner. However, anaerobic microbial species (both bacteria and fungi) have evolved a different mechanism which consists of a scaffolding protein and many bound cellulytic enzymes. This large enzyme complex, known as cellulosomes, is extracellular in nature. Cellulosomes are multi-enzyme complexes consisting of various catalytic domains, carbohydrate binding modules and scaffolding units that enable them to form multiple enzyme-substrate complexes. The multiple enzyme embedded model works synergistically to depolymerize cellulosic material [41].

Enzymes in cellulosomes are spatially arranged in a manner that facilitates the efficient conversion of substrates in a sequential manner. This systematic organization is very distinct and increases the overall efficiency of the hydrolysis process by ensuring that the cascading enzyme reactions channel intermediates between consecutive enzymes thereby avoiding competition with other reactions taking place in the cell [150]. Typical cellulosomes consist of a cohesin–dockerin system of arrangement: cohesins (type 1) are structural scaffold protein that take part in specific, affinity-based interactions with dockerin (type I) which is bound to an assembly of multiple enzymes in a spatially compatible manner. The cohesin–dockerin complex is linked to a scaffolding which typically consist of 26 different cellulosomal units which typically forms the carbohydrate binding module. This unit is connected to the surface of the cell by means of a type II cohesin–dockerin interaction. This makes this enzyme-complex system as effective as crucial cellular biosynthesis models such as Kreb’s cycle [65].

The application of cellulosomes in the conversion of cellulosic materials into fermentable sugars is surging momentum. To determine the effectiveness of cellulosomes as an enzyme system for hydrolysis of cellulose, imperative studies must be conducted. ‘Cellulosomeplus’ was an ambitious project organized by the European Union to create designer cellulosomes to achieve high hydrolysis efficiency of cellulose to augment the shit towards renewable, efficient and inexpensive sources for biotechnological processes [151].

#### 4.2.5. Control of a Fermentation Processes

Along with advances in fermentation technologies, studies have been conducted at lab-pilot scales employing different bacterial and fungal strains to determine the efficacy of enzyme production using lignocellulosic biomass [152]. Several studies have shown a rejuvenated interest in solid-state fermentation for the production of various enzymes. Many of the attributes of agro-industrial residues are especially suited to this form of culture and can yield significant benefits in terms of sustainability. The latter include environmental friendliness and significant reduction in production costs. Mass and energy balances are crucial parameters related to the control of a fermentation processes [153]. The complexity associated with the heat and mass transfer during solid-state fermentation is a major hurdle that has restricted its application in industrial processes [154]. However, recent advances made in this technology by Zhang et al. (2017) suggests that a factor termed the mechanical index (Imp) can be used as an aid to characterize the physical properties of the medium, permitting informed culture medium development and imparting a higher level of process control to the fermentation [154]. Imp is a product of physical attributes of the solid medium such as resilience, cohesiveness, and springiness. During various stages of the fermentation Imp can be correlated (positively and negatively) with thermal conductivity, water retention, gas permeability, thermal diffusivity and biomass content, all of which varies throughout the fermentation process. These physical properties directly influence the heat and mass transfer, which in turn determines the efficiency of the fermentation. Imp could thus be useful in the determination of physical properties of the medium, as well as permitting tighter control of solid-state culture.

## 5. Conclusions

Lignocellulosic food industry waste is the cheapest and most easily available form of carbohydrates for valorisation and subsequent value addition. Both bacterial and fungal species can be deployed for production of various enzymes using agricultural wastes. Pre-treatments that enable high saccharification rates at lower enzyme loadings can further improve the economics of enzyme production. While solid-state fermentation triumphs over submerged fermentation methods by mimicking the natural conditions that favour fungal growth and higher enzyme yields, further studies need to be performed to efficiently scale up the process. However, drawbacks in bioreactor design currently limits heat and mass transfer within such systems. Parameters such as Imp might be useful in commercially scaling up a solid-state fermentation process for enzyme production. An extensive analysis of several studies on the use of inexpensive media components for enzyme production reveals that lignocellulosic food waste shows much promise and can be utilized as a carbon source in mainstream upscale fermentation processes.

## Figures and Tables

**Table 1:** Chemical composition (%) of important agro-industry residues.

Agro-Industry Residue	Carbohydrates	Crude Fibre	Ash	Pectin	Fat	Protein	Lignin	Ref.
Sugarcane Bagasse	66.48 ± 2.68	-	8.80 ± 0.02	-	-	2.3	17.79 ± 0.62	[8]
Rice Bran	14.1 ± 1.1	26.9	3.4–8.1	-	30.4 ± 0.9	38.2 ± 2.3	25.63	[9]
Wheat Bran	56.8	33.4–63.0	3.9–8.10		3.5–3.9	13.2–18.4	5.6	[10]
Spent Coffee Waste	55.53 ± 0.85	60.46 ± 2.2	1.30 ± 0.10	-	2.29 ± 0.30	17.44 ± 0.10	23.90 ± 0.30	[11]
Brewer’s spent grain	79.9 ± 0.5	3.3 ± 0.1	7.9 ± 0.1	-	0.0 ± 0.0	2.4 ± 0.2	30.48 ± 0.8	[12]
Cassava peel	75.5 ± 1.2	11.2 ± 0.6	2.4 ± 0.2	-	3.1 ± 0.1	1.7 ± 0.1	1.92 ± 0.07	[13]
Apple Pomace	48.0–62.0	-	4.7–51.1	-	-	3.9–5.7	23.5	[14]
Crude Olive Pomace	34.8 ± 0.9	-	6.6 ± 0.5	-	16.65 ± 0.09	0.4 ± 1.0	43.2 ± 0.5	[15]
Banana peel	79.0 ± 0.5	9.3 ± 0.1	2.7 ± 0.0	-	3.0 ± 0.2	0.6 ± 0.1	6.4–9.6	[13]
Citrus peel	30	-	1.7	14.4	-	7.9	1.0	[16]

**Table 2:** Price of some of the commercially important cellulytic enzymes [18].

Brand Name	Product	Quantity	Price (in €)
1,4-α-d-Glucan glucohydrolase AMG 300L^™^ Exo-1,4-α-glucosidase Glucoamylase	Amyloglucosidase from *Aspergillus niger* ≥260 U/mL, aqueous solution	50 mL	127.00
1,4-α-d-Glucan-glucanohydrolase Fungamyl^®^ 800 L	α-Amylase from *Aspergillus oryzae* aqueous solution, ≥800 FAU/g	50 mL	95.00
1,6-α-d-Glucan 6-glucanohydrolase Dextranase Plus L	Dextranase from *Chaetomium erraticum*	50 mL	85.50
D-xylose ketol-isomerase Sweetzyme^®^ IT Extra	Glucose Isomerase from *Streptomyces murinus* ≥350 U/g	50 G	248.00
Carezyme 1000L^®^	Cellulase from *Aspergillus* sp. aqueous solution	50 mL	92.50
Lactase Lactozyme^®^ 2600 L	β-Galactosidase from *Kluyveromyces lactis* ≥2600 units/g	50 mL	96.50
Pectinex Ultra Clear^®^	Pectinase from *Aspergillus aculeatus*	50 mL	71.00
Pentopan Mono BG^®^	Xylanase powder, ≥2500 units/g, recombinant, expressed in *Aspergillus oryzae*	50 G	297.00
Promozyme^®^ D2 Pullulanase microbial	Pullulanase microbial	50 mL	78.50
